# A clinical appraisal of the Greiner G300 clinical chemistry analyser

**DOI:** 10.1155/S1463924680000612

**Published:** 1980

**Authors:** M. J. Hallworth, S. G. Archer, J. G. Lines

**Affiliations:** 1 Department of Clinical Biochemistry Addenbrooke's Hospital Hills Road Cambridge CB2 2QR UK; 2 Department of Clinical Chemistry William Harvey Hospital Ashford Kent TN24 OLZ UK


REFERENCES
[1] Tiffany, T. O., Morton, J. M., Hall, E. M. and Garrett, A. S., *Clinical Chemistry*, 1974, **20**, 476.
[2] Ziegenhorn, J., *Clinical Chemistry*, 1975, **21**, 1627.
[3] Wentz, P. W., Cross, R. E. and Savory, J., *Clinical Chemistry*, 1976, **22**, 188.
[4] Grossman, S. H., Mollo, E. and Ertingshausen, G., *Clinical Chemistry*, 1976, **22**, 1310.
[5] Wakayama, J. E. and Swanson, J. R. *Clinical Chemistry*, 1977, **23**, 223.
[6] Chong-Kit, R. and McLaughlin, P., *Clinical Chemistry*, 1974, **20**, 1454.
[7] Bucolo, G. and David, H., *Clinical Chemistry*, 1973, **19**, 476.
[8] Wolf, A. V., Brown, M. G. and Prentiss, P. G. in 'Handbook of Chemistry and Physics' Ed. Weast, R. C., CRC Press, Cleveland, Ohio, D-206.
[9] Stinshoff, K., Weisshaar, D., Staehler, F., Hesse, D., Gruber, W. and Steiner, E., *Clinical Chemistry*, 1977, **23**, 1029.
[10] Lindley, D. V., *Supplement Journal Royal Statistical Society*, 1947, **9**, 218.
[11] Massey, F. J., *Journal American Statistical Association*, 1951, **46**, 68.
[12] Frank, J. J., Bermes, E. W., Bickel, M. J. and Watkins, B. F., *Clinical Chemistry*, 1978, **24**, 1966.

### A clinical appraisal of the Greiner G300 clinical chemistry analyser

**M.J. Hallworth and S.G. Archer**
*Department of Clinical Biochemistry, Addenbrooke's Hospital, Hills Road, Cambridge CB2 2QR.*

**and J.G. Lines**
*Department of Clinical Chemistry, William Harvey Hospital, Ashford, Kent TN24 0LZ.*

### Introduction

The demands on clinical chemistry laboratories over the last two decades have grown so rapidly that automated multiple analysis upon single samples is now the only practicable method of providing a routine clinical chemistry service in all but the smallest hospitals. A large variety of equipment is commercially available for performing this task rapidly and economically, whilst maintaining high standards of analytical quality. Any new instrument must therefore equal or better the established standards of precision, and must use analytical methods producing results as close as possible to reference methods for each analyte.

The work which follows sets out to determine the precision and accuracy of the Greiner G300 analyser in routine use and to examine both the analytical capability of the instrument and also the contribution it can make towards handling the workload of the clinical chemistry laboratory.

The Greiner G300 analyser is a third-generation instrument, which essentially comprises up-dated modifications of the Greiner Selective Analyser (GSA II) which has been available for six years. In the United Kingdom, the GSA II was first formally evaluated by Skinner and Wilding [I] in 1975, and a more comprehensive evaluation has recently been produced by Carlyle *et al* [2] who made their study over a considerable period of routine use.

The present evaluation was planned as a rapid assessment of the clinical utility of the new G300. Following exhibition at the 3rd European Congress of Clinical Chemistry (Brighton, June 1979), a prototype instrument was transported to Cambridge and was operational for this clinical trial the day after arrival. Over the next five days a series of experiments designed to rapidly assess the performance of the G300 under routine conditions was performed.

### Instrument description

The instrument is a discrete analytical system capable of performing up to 30 different analyses on a single specimen. Methods for 20 analyses are already fully documented, and the repertoire is being extended by further tests as they are adapted to the G300 from the GSA II.

The G300 is physically smaller than the GSA II and has improved process control facilities. The number of analyses on each specimen and the rate of analysis remain the same, but the handling of emergency and paediatric samples is faster and more convenient than on the GSA II. A washing cycle for process tubes is a new feature of the G300 and contrasts advantageously to the disposable process tubes used by the GSA II. Carlyle *et al* [2] found that the cost of process tubes considerably exceeded the cost of reagents for most tests, therefore the economic saving is appreciable.

The G300 comprises a 30 place sample carrier and sampling unit, a conveyor belt system on which the process tubes used for reactions are supported and moved, and a photometer unit with associated electronics. The analysis time from sampling to measurement of the final optical density is 13.4 minutes. The reaction time is variable between 36 seconds and 12.6 minutes according to the position of the reagent dispensers within the incubator unit, and the optical density may be read at the wavelength of any one of eight mercury lines, between 334 and 623 nm. The same sample can be dispensed into a pair of process tubes, and up to four different reagents added to each tube. In this manner blank measurements can be determined when required. The operator selects the tests to be performed on each specimen by means of a keyboard next to the sample tray. Commonly-requested groups of analyses (biochemical profiles) can be selected by means of a single key, and some examples of profiling rates are shown in Table 1. It is possible to introduce any urgent investigation into the middle of the batch of other analyses, or to perform only a few necessary tests on a small sample of serum, while performing a wider profile on the remainder of the batch. Emergency samples are placed in the next available space in the sample tray, the operator identifies the sample as an emergency, and keys in the test required: the instrument then positions the emergency samples under the sampling unit, samples sufficient serum for the tests required and then returns to its previous position in the batch of routine analyses.

Although a flame photometer is available for the G300, it was not supplied with the instrument and therefore not evaluated.

The sampling unit has a level sensor, in order that the sample aspirating tube always dips approximately 2 mm below the surface of the liquid in the sample cup. Centrifuged blood samples with the cells still in the tube can be introduced directly onto the machine, provided appropriate sample tubes are used. Since no transfer of serum to other containers is required mistakes in specimen identification are reduced.

The instrument and its methods are designed to work on an absolute basis, calculating results from pre-set parameters and the molar absorptivity of the compound formed when purified standard substrates are reacted in the G300. Continuous use of controls and standards is not required. The instrument did not need recalibration during the five working days that it was evaluated in this laboratory. However, factors can be adjusted if required via the keyboard, but this is only usually necessary on changing batches of certain reagents. All results are calculated according to the general formula:

$$C = (A_2 - A_1) \times \frac{1}{E} \times \frac{FV}{SV} \times \frac{1}{t} \times D$$

where,

C = concentration
E = molar absorptivity of chromogen obtained from purified standard
FV = final volume
SV = sample volume
$A_2$ = test absorbance
$A_1$ = blank absorbance
D = dilution
t = reaction time

It is important to view the precision data and the speed of the analyser in the light of the knowledge that all specimens are patient specimens, and recalibration every tenth specimen is unnecessary. Throughput is therefore improved by approximately 11% in relation to using continuous-flow instruments.

**Table 1. Analysis rate of G300 for commonly requested "profiles"**

| Tests Requested | Rate (specimen/hour) | Total Analytical time for: 1 sample (min) | Total Analytical time for: 10 samples (min) |
|---|---|---|---|
| Cholesterol + triglyceride | 150 | 13.8 | 16.4 |
| Sodium, potassium, glucose, total $CO_2$, urea, creatinine. | 43 | 14.8 | 27.4 |
| Calcium, phosphate, total protein, ALT, albumin, bilirubin, alkaline phosphatase. | 30 | 15.4 | 33.4 |

### Analytical methods

The G300 was evaluated when performing assays for albumin, alkaline phosphatase, calcium, creatinine, glucose, magnesium, total bilirubin, total $CO_2$, total protein, triglyceride and urea. Brief details of the methods employed, and those used for the comparative accuracy study are shown in Table 2. All of the methods used were recommended by the manufacturer of the instrument concerned (Greiner Electronics Ltd., Technicon Instruments Corporation and Pye Unicam Ltd.) and all reagents except those for the analysis of glucose (Boehringer Corporation) and triglyceride (Dow Diagnostics for the G300, BDH Chemicals for continuous flow) were also supplied by the equipment manufacturer.

### Appraisal protocol

The study was designed to give a rapid assessment of the precision, carryover, comparative accuracy and general performance of the G300 in routine use. Precision has been studied at varying levels of analyte concentration for eleven determinations, and comparative accuracy has been examined by comparison of results of patient and control samples analysed on the G300 with results given by the methods

**Table 2. Methods employed on G300 and AutoAnalyser during evaluation**

| Analysis | Greiner G300 method | Comparison method: Equipment | Comparison method: Method |
|---|---|---|---|
| Albumin | Bromocresol green (36 sec.) (Gustaffson, [3]) | Technicon SMA Plus | Bromocresol green (end point) (Doumas *et al*, [4]) |
| Alkaline Phosphatase | 4-nitrophenyl phosphate (37°) (Kuffer & Richterich [5]) | Technicon SMA Plus | 4-nitrophenyl phosphate (37°) (Morgenstern, [6]) |
| Calcium | Cresolphthalein complexone (Kuffer & Degiampietro, [7]) | Technicon SMA Plus | Cresolphthalein complexone (Gittelman, [8]) |
| Creatinine | Rate reaction/alkaline picrate (Bartels & Bohmer, [9]) | Technicon SMA II | End point alkaline picrate (Chasson *et al*, [10]) |
| Glucose | Glucose oxidase/PAP (Trinder, [11]) | Technicon SMA II | Glucose oxidase/3-MBT (Gochman & Schmitz, [12]) |
| Magnesium | Calmagit (Khayam-Bashi *et al*, [13]) | Pye-Unicam SP90 | Atomic absorption (Dawson & Heaton, [14]) |
| Total Bilirubin | 2, 4 dichloroaniline (Kuffer & Degiampietro, [15]) | Technicon SMA Plus | Sulphanilic acid/caffeine (Gambino & Schreiber, [16]) |
| Total $CO_2$ | PEP carboxylase/fast violet B (Forrester *et al*, [17]) | Technicon SMA II | Gas dialysis/cresol red (Skeggs & Hochstrasser, [18]) |
| Total Protein | Biuret (Hoffman & Richterich, [19]) | Technicon SMA Plus | Biuret (Bigat & Saifer, [20]) |
| Triglyceride | Fully enzymatic (Stavropoulos & Crouch, [21]) | Technicon AA II | Dihydrolutidine derivative (Kessler & Lederer, [22]) |
| Urea | Urease/Berthelot (Richterich & Kuffer, [23]) | Technicon SMA II | Diacetyl monoxime (Marsh *et al*, [24]) |

currently used at Addenbrooke's Hospital, based primarily on continuous flow equipment.

### Precision

The overall precision of the analytical system was assessed in a series of three experiments: within-batch precision was assessed by repeated consecutive analysis of the same materials (four control sera with different concentrations of each analyte, see Table 4) for each analyte under evaluation; between batch precision was assessed for most analytes by blind duplicate analyses of up to 150 patient samples on the G300. The between-batch precision was further assessed for four of the analytes selected for evaluation by assaying a serum pool four or five times on each of the five days of the study.

### Accuracy

Results obtained from the G300 for each of seven determinations on between 50 and 150 patient samples were compared with results obtained from Technicon SMA II and SMA 12/60 systems. Analysis on the G300 followed analysis on Technicon systems, normally the following day but in some cases a week-end intervened between the two analyses. Total bilirubin and total $CO_2$ were excluded from this part of the evaluation in view of the instability of these constituents during storage. Insufficient data was available to make valid comparisons for magnesium and triglyceride.

The methods used for comparison are not offered as reference methods. However, this laboratory participated in the UK National Quality Control Scheme and in the Wellcome Quality Control Scheme. The variance indices of this laboratory for the National Quality Control Scheme for the week in which the evaluation was performed were as follows:–

| | | | |
|---|---|---|---|
| urea | : 53 | creatinine | : 17 |
| glucose | : 33 | total bilirubin | : 45 |
| calcium | : 72 | total protein | : 21 |
| phosphate | : 52 | albumin | : 33 |

The Overall Mean Running Variance Index Score for this laboratory was 48, which contrasts favourably with the national average of 60 for the same size of laboratory.

In an attempt to obtain further independent assessment of accuracy, two batches of Technicon ILCS sera (A4SO97 and B4S141, Technicon Instruments Ltd. Basingstoke, Hants) were each assayed 15 times on the G300, and the mean result obtained for each constituent was compared with the overall mean value for that constituent found by all the laboratories participating in the Technicon Inter-Laboratory Quality Control Scheme.

### Carryover

Since the G300 makes a separate sampling motion for each determination on a particular specimen, carryover between specimens can only occur between the final sample removed from the first specimen, and the first determination on the succeeding specimen. Carryover is therefore maximised if a determination requiring a high volume of serum (eg creatinine, 60 μl) is followed directly by water or a serum with low levels of all analytes. Carryover between successive specimens for the same determination is not a problem when multiple analyses are performed on the G300, because a number of sampling cycles separates the same determination on succeeding specimens. Carryover by contamination of tubes or cuvettes is restricted as both are washed twice and dried before reuse.

A series of experiments was performed with serum containing high levels of all analytes, followed by two or three cups filled with distilled water which were assayed for all constituents. Carryover was not found to be significantly different from zero for any of the analyses undertaken. (Accordingly detailed results are not shown but are available if required). Similar findings for the GSA II were reported by Skinner & Wilding [1] and Carlyle *et al* [2].

**Table 3. Frequency of outlier results**

| Analysis | Number of outliers | Total number | % |
|---|---|---|---|
| Creatinine | 64 | 400 | 16.0 |
| Magnesium | 3 | 90 | 3.7 |
| Total bilirubin | 20 | 197 | 10.2 |
| Total protein | 13 | 194 | 6.7 |

### Frequency of outlier (gross error) results

As all analyses on the G300 were performed on sera with known values for all constituents or on patient material assayed in duplicate, grossly deviant results were readily identified and were looked for. The frequency of occurrence of such outliers was calculated for each determination and is shown in Table 3 for the four analytes in which this problem occurred. Most of these outliers can be recognised by the system and excluded (see discussion) before result release or production.

### Results

### Precision

The estimates of within-batch and between-batch precision, and the precision of replicate analyses are shown in Tables 4 and 5 after exclusion of the outliers. Total bilirubin and total $CO_2$ have been excluded from the tables because these constituents in sera alter during the period between replicate analyses.

### Accuracy

The results of linear regression analysis as applied to the data from the G300 and Technicon systems on patient sera are shown in Table 6. Figures 1 and 2 illustrate the best correlation (urea) and the worst correlation (albumin). The comparison between the G300 results (mean of 15 analyses) for Technicon ILCS sera A4S097 and B4S141 and the published overall mean result from all laboratories is shown in Table 7.

### Discussion

### Precision

A number of workers have attempted to define acceptable or target standards of precision for clinical laboratories. Tonks [26] suggested that the analytical standard deviation should not exceed 25% of the normal range, leading to an allowable co-efficient of variation (CV) calculated from the formula:

$$Allowable\ analytical\ CV = \frac{0.25\ width\ of\ normal\ range}{Mean\ normal\ value} \times 100$$

and further suggested that the maximum allowable analytical CV be 10%. Cotlove *et al* [27] have postulated that the analytical coefficient of variation should be less than half the composite biological coefficient of variation, defined as the combined variance due to within and between person variation. These criteria are shown in Table 8 for various determinations, together with the experimentally-determined within-batch and between-batch coefficients of variation at the appropriate level of the analyte concentration. The between-batch precision attained by the G300 meets Tonks' criteria where data is available, and betters the more stringent Cotlove criteria for all analytes except calcium, magnesium and total $CO_2$.

Many laboratories find difficulty in achieving the Cotlove CV for calcium (1.6%). The G300 value of 5.3% for within-batch calcium CV is seriously worse than the Cotlove criteria allow and is unacceptable for clinical analysis at this level. Rather better values are obtained at other levels of calcium concentration (see Table 4).

The within-batch precision estimates in Table 4 were performed with the G300 operating as a multiple analyser for all eleven tests, and thus successive determinations for a particular test are not consecutive, but are separated by ten other sampling motions, each requiring different volumes of the control serum.

**Table 4. G300 within-batch precision**

| Analysis & units | Material (see below) | Mean value | SD | CV (%) | Analysis & units | Material (see below) | Mean value | SD | CV (%) |
|---|---|---|---|---|---|---|---|---|---|
| Albumin g/l | B | 23.6 | 0.34 | 1.5 | Total Bilirubin | B | 12 | 0.5 | 4.2 |
| | D | 30.6 | 0.36 | 1.2 | μmol/l | D | 42 | 1.0 | 2.4 |
| | A | 41.1 | 0.89 | 2.2 | | A | 49 | 1.0 | 2.1 |
| | C | 43.9 | 1.1 | 2.4 | | C | 107 | 1.7 | 1.6 |
| Alkaline | B | 63 | 1.9 | 3.0 | Total $CO_2$ mmol/l | B | 18.0 | 0.74 | 4.1 |
| phosphatase u/l | D | 174 | 4.0 | 2.3 | | A | 23.2 | 0.96 | 4.1 |
| | A | 195 | 4.1 | 2.1 | | D | 26.2 | 0.58 | 2.2 |
| | C | 324 | 6.0 | 1.9 | | C | 29.1 | 1.16 | 4.0 |
| Calcium mmol/l | B | 1.73 | 0.155 | 9.0 | Total protein g/l | B | 43.0 | 0.92 | 2.1 |
| | D | 2.29 | 0.065 | 2.8 | | A | 73.0 | 1.24 | 1.7 |
| | A | 2.53 | 0.134 | 5.3 | | D | 74.5 | 0.91 | 1.2 |
| | C | 2.92 | 0.107 | 3.7 | | C | 84.6 | 3.20 | 3.8 |
| Creatinine μmol/l | B | 117 | 4.6 | 3.9 | Triglyceride | B | 0.48 | 0.020 | 4.2 |
| | D | 158 | 6.6 | 4.2 | mmol/l | D | 0.60 | 0.015 | 2.4 |
| | A | 462 | 8.7 | 3.4 | | C | 1.00 | 0.028 | 2.8 |
| | C | 700 | 24.0 | 1.9 | | A | 2.74 | 0.055 | 2.0 |
| Glucose mmol/l | B | 5.0 | 0.20 | 4.1 | Urea mmol/l | B | 1.9 | 0.09 | 4.6 |
| | D | 6.2 | 0.12 | 2.0 | | A | 8.4 | 0.17 | 2.0 |
| | A | 12.5 | 0.20 | 1.6 | | D | 10.0 | 0.18 | 1.8 |
| | C | 20.0 | 0.61 | 3.0 | | C | 12.5 | 0.17 | 1.3 |
| Magnesium mmol/l | B | 0.35 | 0.045 | 13.0 | | | | | |
| | A | 0.45 | 0.051 | 11.4 | | | | | |
| | C | 0.66 | 0.072 | 10.9 | | | | | |
| | D | 0.91 | 0.037 | 4.1 | | | | | |

A. Technicon Reference Serum, batch B8G637
B. Technicon ILCS quality control serum, A4S097
C. Technicon ILCS quality control serum, B4S141
D. Wellcomtrol I unassayed serum, lot K6314

**Table 5. G300 between-batch precision**

(a) Standard deviation of duplicate analyses (see text)

| Analysis & units | Number of pairs (n) | SD of duplicates | Range of values |
|---|---|---|---|
| Albumin g/l | 53 | 0.74 | 17 – 43 |
| Alkaline phosphatase U/l | 53 | 4.06 | 67 – 921 |
| Calcium mmol/l | 53 | 0.05 | 1.16 – 3.26 |
| Creatinine μmol/l | 101 | 9.58 | 63 – 1052 |
| Glucose mmol/l | 145 | 0.43 | 3.2 – 17.6 |
| Total protein g/l | 45 | 0.97 | 53 – 81 |
| Urea mmol/l | 143 | 0.18 | 1.3 – 45.0 |

(b) Precision over 5 days (n = 21). Material A. (see Table 4)

| Analysis & units | Mean value | SD | CV |
|---|---|---|---|
| Creatinine μmol/l | 156 | 11.4 | 7.3 |
| Glucose mmol/l | 6.3 | 0.26 | 4.1 |
| Total $CO_2$ mmol/l | 24.7 | 1.48 | 6.0 |
| Urea mmol/l | 10.0 | 0.21 | 2.1 |

(Robinson, [25])

Standard deviation = $\sqrt{\frac{\Sigma d_i^2}{2n-1}}$

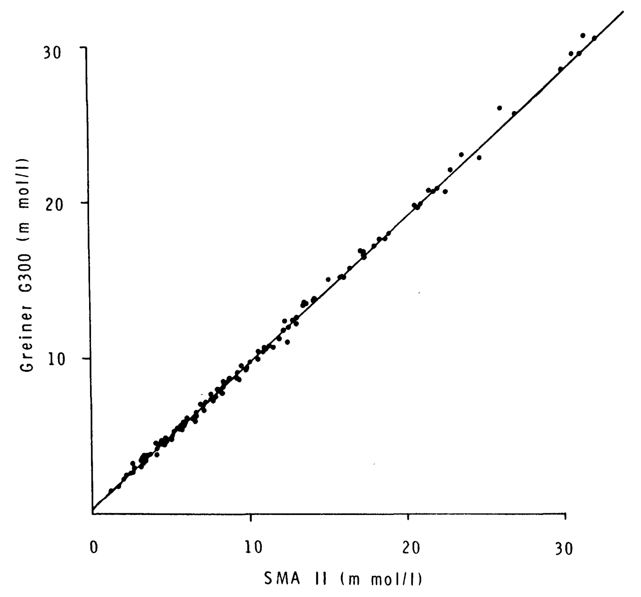

*Figure 1. Correlation of urea results – Greiner G300 versus SMA II.*

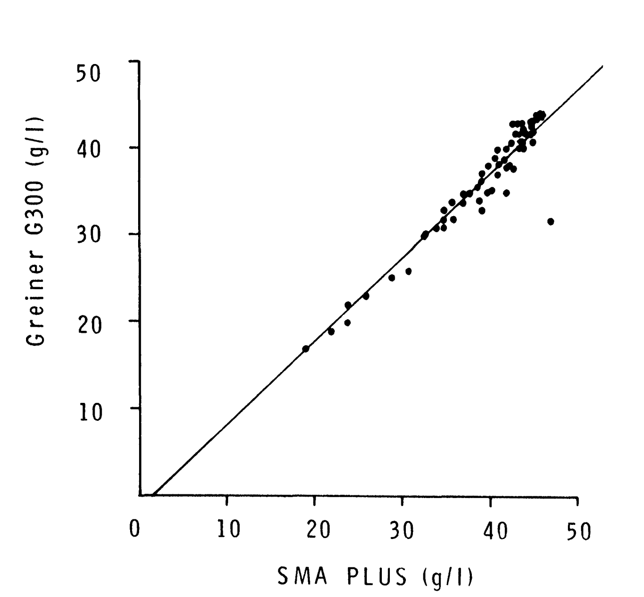

*Figure 2. Correlation of albumin results – Greiner G300 versus SMA Plus.*

The standard deviation of duplicate analyses has been determined for seven determinations (Table 5a). Robinson [25] states that this parameter usually represents a good estimate of the between-batch standard deviation, although it is clearly an average estimate across a range of analytical concentration. Between-batch precision estimates determined over five days give similar values (Table 5b).

The precision of the G300 is clinically acceptable for all determinations, with the possible exception of calcium. Precision is better than the SMA system used for comparison for alkaline phosphatase and urea, similar for total protein and albumin, and worse than the SMA systems for calcium, creatinine, glucose and total $CO_2$.

### Accuracy

The results of the correlation between the G300 and SMA analysers (Table 4) show that the determinations of alkaline phosphatase, creatinine, glucose and urea correlate well (r>0.98). Those for albumin, calcium and total protein are less ideal, but still greater than 0.94.

The gradients of the regression lines represent lower values given by the G300 for creatinine, urea, albumin and calcium. The greater specificity of the G300 determinations for creatinine (kinetic versus end point Jaffé), urea (enzymatic versus diacetyl monoxime) and albumin (short versus long reaction with BCG) is likely to account for much of this and the Greiner result may more nearly reflect the reference method result. Calcium methods are the same on both machines, so that an explanation of the difference here probably has to be based on dialysis by one machine and not by the other.

Values for alkaline phosphatase are significantly higher on the G300 than on the SMA 12/60 (gradient of regression line = 1.43). The instruments use the same substrate and reaction temperature (4-nitro phenyl phosphate, 37°), but in different buffer systems (diethanolamine for the G300, 2-amino 2-methyl propan-1-ol for the SMA 12/60). Methods using the G300 combination of substrate and buffer have been reported to give elevated values for alkaline phosphatase (End of Period Report, Wellcome Group Quality Control Programme, Wellcome Reagents Ltd., March 1979), but this should not affect the interpretation of results when the reference range is properly defined.

The G300 results compare reasonably well with the consensus values reported for Technicon ILCS sera A4S097 and B4S141 (Table 7). All G300 results lie within two standard deviations of the consensus value, with the exception of alkaline phosphatase, creatinine and urea. The elevated alkaline phosphatase results have already been attributed to the use of diethanolamine buffer, and the discrepant urea results may be explained by the fact that the diluent used for Technicon ILCS sera contains ammonia, which interferes with the urease/phenol/hypochlorite method for urea on the G300.

**Table 6. Correlation of results – G300 versus Technicon SMA**

| Analysis & units | Number | Correlation coefficient | Gradient | Intercept |
|---|---|---|---|---|
| Albumin g/l | 54 | 0.947 | 0.967 | −2.01 |
| Alkaline phosphatase μ/l | 54 | 0.986 | 1.430 | 61.4 |
| Calcium mmol/l | 54 | 0.968 | 0.963 | −0.033 |
| Creatinine μmol/l | 140 | 0.997 | 0.896 | 29.4 |
| Glucose mmol/l | 150 | 0.994 | 0.996 | −0.324 |
| Total protein g/l | 52 | 0.966 | 0.996 | 1.54 |
| Urea mmol/l | 146 | 0.998 | 0.941 | 0.193 |

**Table 7. Comparison of G300 results with consensus values for Technicon ILCS sera (A = serum A4S097, B = serum B4S141)**

| Analysis & units | | G300 (Mean of 15 results) | Consensus Mean | Consensus SD | Deviation (SD units) from consensus value* |
|---|---|---|---|---|---|
| Albumin g/l | A | 23.7 | 25.6 | 1.30 | −1.5 |
| | B | 43.9 | 45.7 | 1.93 | −0.9 |
| Alkaline Phosphatase μ/1 | A | 63.5 | 47 | 4.43 | +3.7 |
| | B | 324 | 199 | 15.62 | +8.0 |
| Calcium mmol/l | A | 1.75 | 1.79 | 0.07 | −0.8 |
| | B | 2.92 | 2.95 | 0.09 | −0.3 |
| Creatinine μmol/l | A | 117 | 102.7 | 8.23 | +1.7 |
| | B | 700 | 788.9 | 33.82 | −2.6 |
| Total $CO_2$ mmol/l | A | 18.0 | 18.0 | 1.44 | 0 |
| | B | 29.0 | 30.8 | 2.11 | −0.8 |
| Total bilirubin μmol/l | A | 12.1 | 14 | 1.86 | −1.0 |
| | B | 107 | 116 | 8.48 | −1.0 |
| Total protein g/l | A | 43.0 | 42.1 | 1.36 | +1.36 |
| | B | 84.6 | 80.9 | 2.93 | +1.3 |
| Urea mmol/l | A | 1.95 | 4.7 | 0.31 | −8.8 |
| | B | 12.5 | 28.5 | 0.91 | −17.5 |

**This represents the difference between the G300 mean value and the consensus mean value expressed as a fraction of the consensus S.D.*

### Outlier results

As is clear from Table 3, outlier results for creatinine, total bilirubin and total protein occurred with unacceptable frequency on the instrument under test. This problem is compounded when an outlier result is within the linear range of the instrument, because there is no indication on the machine output that a fault has occurred unless a result falls outside pre-defined ranges: outliers which are clinically feasible are thus undetectable without analysing samples in duplicate, which is clearly impractical.

In general, since there is no visual contact with the analytical system for much of the time, it is not easy to pinpoint the cause of random errors. Air bubbles in the photometer cell or incomplete dispensing of a particular reagent are not readily detectable. It is possible to examine the raw optical density data supplied by the photometer, and this is of undoubted value in tracing certain faults, but the possibility of random outliers passing undetected was strong. The more recent production models of the G300 incorporate a photometer system that performs multiple optical density readings on each cuvette with truncation of exceptional values, and hence can detect air bubbles and particulate matter. It is, therefore, possible that the high outlier frequency was unique to this prototype instrument and recent information indicates that present models do not have this problem.

**Table 8. Relation of G300 precision to published criteria (see text)**

| Constituent & approximate level | Greiner G300 CV (%) Within batch | Greiner G300 CV (%) Between batch | Tonks CV (%) | Cotlove CV (%) |
|---|---|---|---|---|
| Albumin 40 g/l | 2.2 | ND | 10.0 | 3.5 |
| Alkaline phosphatase 60 U/l | 3.0 | ND | 10.0 | – |
| Calcium 2.5 mmol/l | 5.3 | ND | 6.0 | 1.6 |
| Creatinine 156 μmol/l | 4.2 | 7.3 | 10.0 | – |
| Glucose 6.0 mmol/l | 2.0 | 4.1 | 10.0 | 4.7 |
| Magnesium 0.9 mmol/l | 4.1 | ND | 8.8 | 3.3 |
| Total bilirubin 15 μmol/l | 4.2 | ND | 10.0 | – |
| Total $CO_2$ 26 mmol/l | 2.2 | 6.0 | 6.0 | 3.1 |
| Total protein 70 g/l | 1.7 | ND | 7.0 | 3.2 |
| Triglyceride 1.0 mmol/l | 2.8 | ND | 10.0 | – |
| Urea 10.0 mmol/l | 1.8 | 2.1 | 10.0 | 7.5 |

ND = not done

### Calibration

The chemical stability of the G300 in the absence of repeated calibration is impressive, and provides the opportunity for considerable reduction of expenditure on calibration material. The long-term stability of calibration in routine use was not assessed here.

### General performance

Obviously a five-day evaluation period is inadequate to indicate a long-term reliability of the G300. However, we were impressed by the ability of the machine to produce results within 24 hours of arrival in the laboratory, having been transported considerable distances by road.

There were no mechanical or electronic problems causing significant down-time during the week in which the instrument was studied.

### Conclusion

The Greiner G300 analyser is an efficient discrete analyser with adequate standards of accuracy and precision. It requires little sample, and all eleven tests discussed in this evaluation can be performed on 400 $\mu$l serum. The manufacturer states that method sensitivity is such that the same analyses could be carried out on a pre-diluted sample of serum, such that an initial 50 $\mu$l only of serum would be required.

The design of the analyser makes it very easy to perform only those tests actually requested by the clinician, with consequent savings of time and reagents. Worklists and batching of tests are unnecessary. Laboratories with good ward liaison could therefore significantly reduce their workload while providing the same amount of useful biochemical information.

The G300 is well suited to paediatric and emergency work, and could easily handle the combined routine paediatric and adult emergency analyses for even large hospitals. The capital cost of the instrument may appear to make it an expensive solution to providing for these analyses, but substantial staff savings would be effected.

Throughput is more than adequate for single analyses but becomes slow in comparison to other equipment if more than five or six analyses per specimen are required.

The G300 should be capable of handling the major analytical workload for laboratories with a total workload of 500,000 requested tests. It was not designed for, and is not suitable for, laboratories committed to a large 'profiling' workload.

With improved elimination of outlier results, the G300 provides a convenient and reliable solution to the problems of smaller hospitals with modest workloads spread over a variety of analyses. It is capable of providing a rapid paediatric and emergency service for even the largest hospitals.

REFERENCES

[1] Skinner, A.G. and Wilding, P., *Annals of Clinical Biochemistry,* 1975, **12**, 182.
[2] Carlyle, J.E., Fleck, A., Hay, G.D., and McIndoe, J., Evaluation of the Greiner Selective Analyser GSA II. Department of Health and Social Security, London.
[3] Gustaffson, J.E.C., *Clinical Chemistry,* 1976, **22**, 616.
[4] Doumas, B.T., Watson, W.A. and Biggs, H.G., *Clinica Chimica Acta,* 1971, **31**, 87.
[5] Küffer, H. and Richterich, R., *Quaderni Sclavo di Diagnostica Clinica e di Laboratorio,* 1973, **9**, 61.
[6] Morgenstern, S., Kessler, G. and Auerbach, J., *Clinical Chemistry,* 1965, **11**, 876.
[7] Küffer, H. and Degiampietro, P., *Clinical Chemistry,* 1975, **21**, 961.
[8] Gitelman, H.J., *Analytical Biochemistry,* 1967, **18**, 521.
[9] Bartels, H. and Böhmer, M., *Clinica Chimica Acta,* 1971, **32**, 81.
[10] Chasson, A.L., Grady, H.T. and Stanley, M.A., *American Journal of Clinical Pathology,* 1961, **35**, 83.
[11] Trinder, P., *Annals of Clinical Biochemistry,* 1969, **6**, 24.
[12] Gochman, N. and Schmitz, J.M., *Clinical Chemistry,* 1972, **18**, 943.
[13] Khayam-Bashi, H., Liu, T.Z. and Walter, V., *Clinical Chemistry,* 1977, **23**, 289.
[14] Dawson, J.B. and Heaton, F.W., *Biochemical Journal,* 1961, **80**, 99.
[15] Küffer, H., and Degiampietro, P., Abstracts 2nd European Congress on Clinical Chemistry, Prague, October 1976.
[16] Gambino, S.R. and Schreiber, S., in "Automation in Analytical Chemistry", Technicon Symposium 1964, Ed. L.T. Skeggs. Mediad, Inc., New York, 1965.
[17] Forrester, R.L., Wataji, L.J., Silvermann, D.A. and Pierre, K.J. *Clinical Chemistry,* 1976, **22**, 243.
[18] Skeggs, L.T. and Hochstrasser, H., *Clinical Chemistry,* 1964, **10**, 918.
[19] Hoffman, J.P. and Richterich, R., *Zeitschrift für Klinishe Chemie und Klinishe Biochemie,* 1971, **8**, 595.
[20] Bigat, T.K., and Saifer, A., *Clinical Chemistry,* 1972, **18**, 630.
[21] Stavropoulos, W.S. and Crouch, R.D., *Clinical Chemistry,* 1974, **20**, 857.
[22] Kessler, G. and Lederer, H., in "Automation in Analytical Chemistry", Technicon Symposium 1965, Ed. L.T. Skeggs. Mediad, Inc., New York, 1966 pp 341-344.
[23] Richterich, R. and Kuffer, H., *Zeitschrift fur Klinishe Chemie und Klinishe Biochemie,* 1973, **11**, 553.
[24] Marsh, W.H., Fingerhut, B. and Miller, H., *Clinical Chemistry,* 1965, **11**, 624.
[25] Robinson, R., "Clinical Chemistry and Automation", 1971, Charles Griffin & Co., London, p.115.
[26] Tonks, D.B. *Clinical Chemistry,* 1963, **9**, 217.
[27] Cotlove, E., Harris, E.K. and Williams, G.Z., *Clinical Chemistry,* 1970, **16**, 1028.

